# Characterization of the inflammatory microenvironment and hepatic macrophage subsets in experimental hepatocellular carcinoma models

**DOI:** 10.18632/oncotarget.27906

**Published:** 2021-03-16

**Authors:** Helena Degroote, Sander Lefere, Astrid Vandierendonck, Bart Vanderborght, Tim Meese, Filip Van Nieuwerburgh, Xavier Verhelst, Anja Geerts, Hans Van Vlierberghe, Lindsey Devisscher

**Affiliations:** ^1^Hepatology Research Unit, Department of Internal Medicine and Pediatrics, Liver Research Center Ghent, Faculty of Medicine and Health Sciences, Ghent University, Ghent, Belgium; ^2^Gut-Liver Immunopharmacology Unit, Department of Basic and Applied Medical Sciences, Liver Research Center Ghent, Faculty of Medicine and Health Sciences, Ghent University, Ghent, Belgium; ^3^Laboratory of Pharmaceutical Biotechnology, Department of Pharmaceutics, Faculty of Pharmaceutical Sciences, Ghent University, Ghent, Belgium

**Keywords:** hepatocellular carcinoma, macrophage subsets, tumor microenvironment, tumor associated macrophages, inflammation

## Abstract

Hepatocellular carcinoma (HCC) is one of the leading causes of cancer-related death worldwide. HCC typically develops on a background of chronic inflammation and fibrosis with tumor associated macrophages (TAMs) playing an important role in chronic inflammation-induced HCC and progression. However, the liver harbors unique macrophages, resident liver Kupffer cells (KCs) and monocyte-derived macrophages (Mo-Mφ), and their contribution to HCC and to the population of TAMs is incompletely known. Here, we characterized the tumor microenvironment and the proportion and transcriptional profile of hepatic macrophages (Mφ) in two commonly used HCC mouse models. A gradually increased expression of inflammatory, immune regulatory, fibrotic and cell proliferation pathways and markers was observed during diethylnitrosamine (DEN)- and non-alcoholic steatohepatitis (NASH)-induced HCC development. The transcriptional phenotypes of isolated hepatic Mφ subsets were clearly distinct and shifted during HCC development, with mixed pro-inflammatory and tumor-promoting expression profiles. There were marked differences between the models as well, with Mφ in NASH-HCC exhibiting a more immunomodulatory phenotype, in conjunction with an upregulation of lipid metabolism genes. Our data show that at least some infiltrated macrophages display expression of pro-tumoral markers, and that Kupffer cells are part of the population of TAMs and enhance tumor progression. These insights are useful to further unravel sequential pathogenic events during hepatocarcinogenesis and direct future development of new treatment strategies for HCC.

## INTRODUCTION

The incidence of hepatocellular carcinoma (HCC) is increasing worldwide, and HCC is amongst the leading causes of cancer death globally. HCC represents about 90% of all cases of primary liver cancer and occurs predominantly in patients with underlying chronic liver disease. Because of the rising incidence of obesity, diabetes and metabolic syndrome, non-alcoholic fatty liver disease (NAFLD) and its progressive inflammatory form, non-alcoholic steatohepatitis (NASH), are projected to become the leading cause of HCC [1].

HCC is considered a classic inflammation-related cancer, whereby inflammation drives carcinogenesis by inducing continuous cycles of tissue damage, necrosis with regeneration and subsequent fibrosis, cell dysplasia and ultimately HCC lesions. The development of tumoral lesions is a multistep process involving different genetic alterations that lead to malignant transformation of hepatocytes. Several rodent models have been used in defining the pathogenesis of HCC and contributed to the current knowledge of HCC. Diethylnitrosamine (DEN) is the most extensively used genotoxic agent for chemically induced HCC. DEN alkylates DNA and induces reactive oxygen species through the activation of the cytochrome P450 in hepatocytes. Transcriptomic analyses showed that the gene expression pattern of the DEN-induced mouse tumors resembles that of a subclass of human HCC with poor prognosis [2–5]. More specifically, the DEN model most closely reflects alcohol-induced HCC in terms of histological and genetic alterations [6]. Due to the increasing incidence of obesity and NAFLD, additional diet-based models have been developed to reliably reproduce the pathological changes observed in human disease. The streptozotocin (STZ) + high-fat or western diet model induces HCC on a background of diabetes, NASH and liver fibrosis. The observations that there were at least 4 nodules with an average tumor growth rate of 150% from 16 to 20 weeks of age, no visible metastasis and preserved liver function suggested that HCC induced in this model is equivalent to more advanced stages B to C of the Barcelona Clinic Liver Cancer staging system for patients [7].

The liver tumor microenvironment (TME) consists of cancer cells surrounded by extracellular matrix elements and different stromal cells such as fibroblasts, endothelial cells and inflammatory cells which secrete a variety of growth factors, cytokines or matrix remodeling enzymes [8–12]. Tumor-associated macrophages (TAMs), representing the predominant type of leukocytes [13], are key players in the TME [14, 15], and their presence is often associated with poor prognosis in HCC patients [16–19]. TAMs promote hepatocarcinogenesis by stimulating tumor cell proliferation, cancer invasion and metastasis, angiogenesis, immune suppression, epithelial-mesenchymal transition and maintenance of stemness [19–27]. In general, macrophages (Mφ) are among the most plastic cells of the hematopoietic system with a polarization spectrum defined by two extremes, the classically activated (M1-like) cells generally exerting pro-inflammatory/anti-tumoral and alternatively activated (M2-like) cells exerting immune suppressive/pro-tumoral functions [14, 20, 21, 28]. In particular, TAMs have served as a paradigm for the plasticity and functional polarization of the mononuclear phagocyte system. Pro-inflammatory Mφ can suppress early HCC tumorigenesis by eliminating cancer cells, but on the other hand also sustain the chronic state of inflammation. During tumor progression, Mφ polarization shifts towards an immune suppressive and pro-tumoral phenotype [20, 21, 23, 27] in response to signals from the tissue microenvironment and stress signals related to inflammation [22, 29, 30].

The liver harbors Mφ of different origins, for which specific markers have become available over the last years, allowing a deeper study of their roles in health and disease. Conventional self-renewing, yolk-sac-derived Kupffer cells (KCs) are the dominant Mφ in the liver sinusoids under homeostatic conditions, where they clear gut-derived signals, inhibit auto-reactive T-cells, and aid in maintaining the hepatic metabolism [31]. A disruption of the steady state by liver injury leads to the secretion of chemokines to attract circulating bone-marrow derived monocytes (Mo) to areas of hepatocyte damage. These infiltrating Mo then differentiate into monocyte-derived Mφ (Mo-Mφ) [32–40]. Indeed, we previously reported that in models of DEN- and NASH-induced HCC, Mo massively infiltrate the liver KCs, whereas KC are partially depleted during chronic liver injury, including end-stage HCC [41]. However, the function of these cells at specific time points, the extent to which these Mφ populations are functionally distinct and how they contribute to HCC development remains to be fully addressed. In this study, we assessed the time-dependent characteristics of the inflammatory micro-environment in the two aforementioned HCC mouse models, with an emphasis on the differential transcriptional profile of liver Mφ subsets and the sequential changes in phenotype during the progression of HCC. The resulting understanding of Mφ function and the implicated immune and metabolic pathways pave the way for targeting these Mφ in the treatment of HCC, which is a field of considerable interest [22, 25, 42–46].

## RESULTS

### Tumor development in the DEN- and NASH-HCC models

The clinicopathological features of HCC development were assessed in the DEN- and NASH-induced HCC model as described in the materials and methods section. Different time points were defined as early, intermediate and end-stage disease according to previously published characteristics of the models. In the DEN model, readily distinguishable nodules of neoplasia were seen at week 25 and week 30 (‘end-stage’). Notably, the percentage of FABP negative sites, number of inflammatory foci and expression of angiogenic markers already increased from week 16 (‘intermediate stage’) [47]. In the NASH-induced HCC model, steatosis is evident at week 6 (‘early stage’), steatohepatitis at week 8, with fibrosis at weeks 8–12 (‘intermediate stage’) and tumor development at week 16 (‘end-stage’) [48].

**Figure 1:**
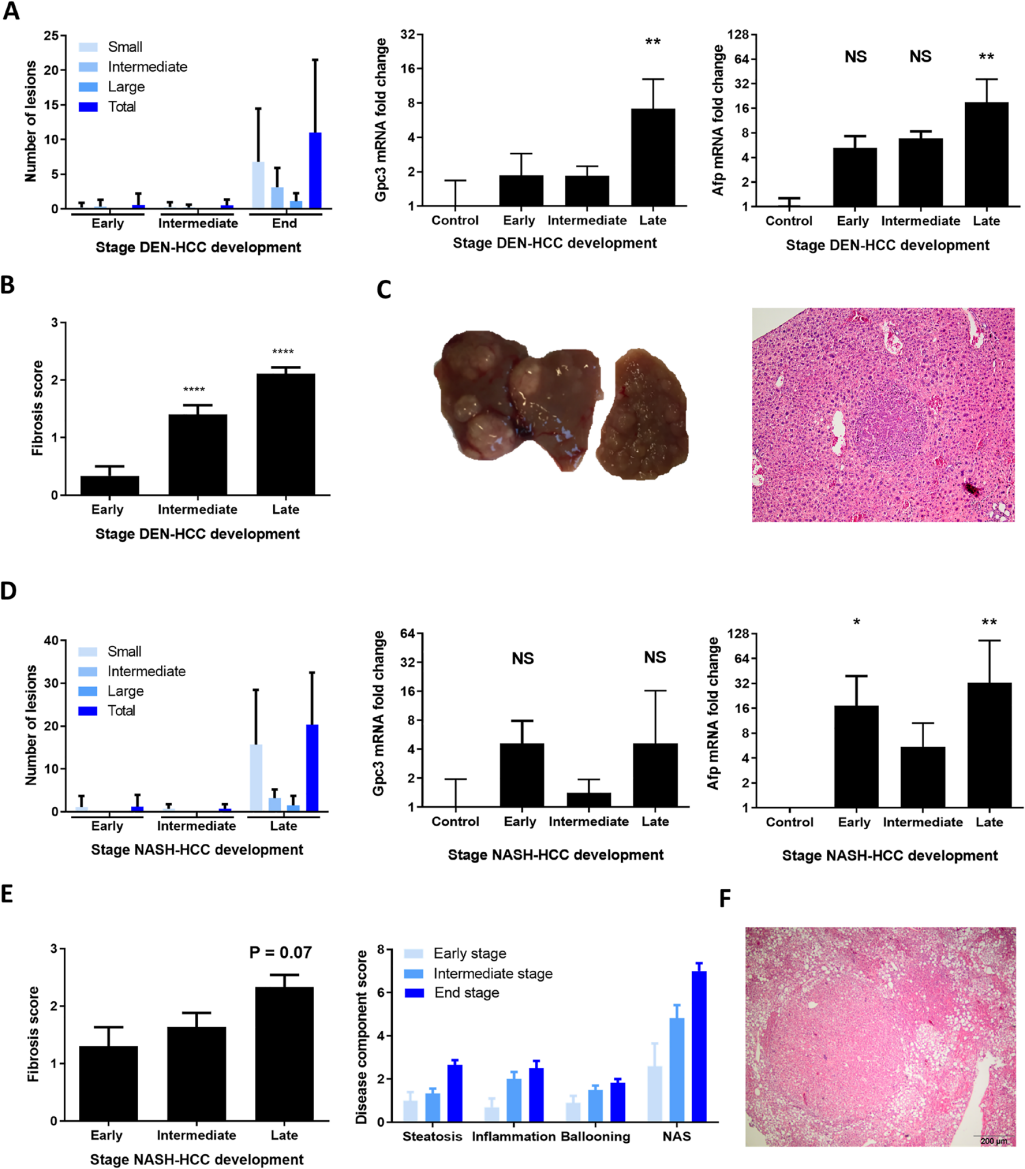
Clinicopathological features of DEN and NASH-induced HCC development. (**A**) Number of liver tumors in the DEN model and mRNA expression of Gpc3 and Afp. (**B**) Fibrosis stage as scored according to the metavir score. (**C**) Representative macroscopic liver image and histological H&E staining (magnification 100x). (**D**) Number of liver tumors in the NASH-HCC model and mRNA expression of Gpc3 and Afp. (**E**) Fibrosis stage as scored according to the NAFLD fibrosis socre and NAFLD activity score with components. (**F**) Representative histological H&E staining, magnification 100x. Variables are presented as mean ± SEM. Multiple comparisons were performed using analysis of variance (ANOVA). ^*^ *P* < 0.05, ^**^ *P* < 0.01, ^***^ *P* < 0.001 compared to control. Abbreviations: ns, non-significant, AFP: alpha-foetoprotein, GPC3: glypican3, NAS: NAFLD activity score. DEN-model: control (*n* = 7), early (*n* = 9), intermediate (*n* = 10) and end-stage (*n* = 10). NASH-model: control (*n* = 5), early (*n* = 10), intermediate (*n* = 12) and end-stage (*n* = 6).

In the DEN-model, small and intermediate nodules were present in few mice at the early and intermediate stage (*n* = 1 and *n* = 2, respectively). In the NASH-model small nodules were present at the early and intermediate stage (*n* = 2 and *n* = 4, respectively), whereas in both models all mice developed tumor nodules at end-stage disease (Figure 1A and 1D). The presence of HCC was confirmed on liver histology and by an increased expression of the HCC markers glypican-3 (Gpc3) and alpha-fetoprotein (Afp), although this did not reach significance for Gpc3 in the NASH-HCC model (Figure 1A, 1C, 1D and 1F). At the late timepoint, mice in both models had progressed to F2 fibrosis as scored on sirius red staining (Figure 1B and 1E). Steatosis, inflammation and ballooning could already be observed at the early stage in the NASH-HCC model, the severity of which gradually increased over time (Figure 1E). Thus, these findings confirm the gradual development of HCC on a background of moderate fibrosis, and, in the STZ-WD model, progressive NASH.

### HCC development is characterized by an altered immunologic and metabolic profile

To further characterize the inflammatory micro-environment, liver tissue of healthy mice and mice at different stages of HCC development was analysed by mRNA sequencing. Gene enrichment mapping of significantly (FDR 0.01) up- or downregulated GO pathways in liver tissue at end-stage HCC development compared to healthy controls was performed.

**Figure 2:**
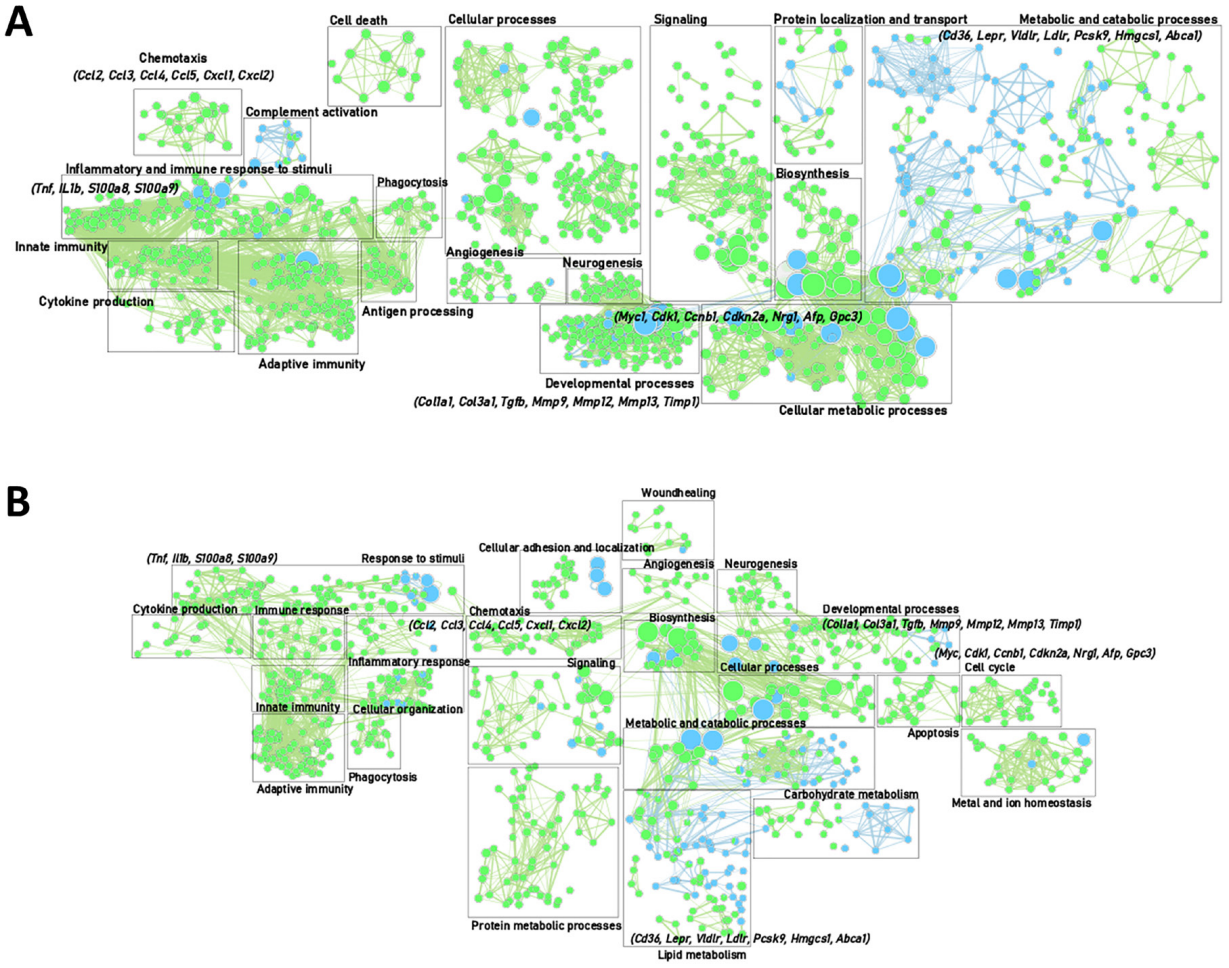
Gene enrichment mapping of significantly (FDR 0.01) up- or downregulated (green and blue, respectively). GO pathways comparing end-stage and controls in the DEN-induced HCC model (**A**) (control (*n* = 4) and end-stage (*n* = 4)) and NASH-induced HCC mode (**B**) (control (*n* = 4) and end-stage (*n* = 4)).

**Figure 3:**
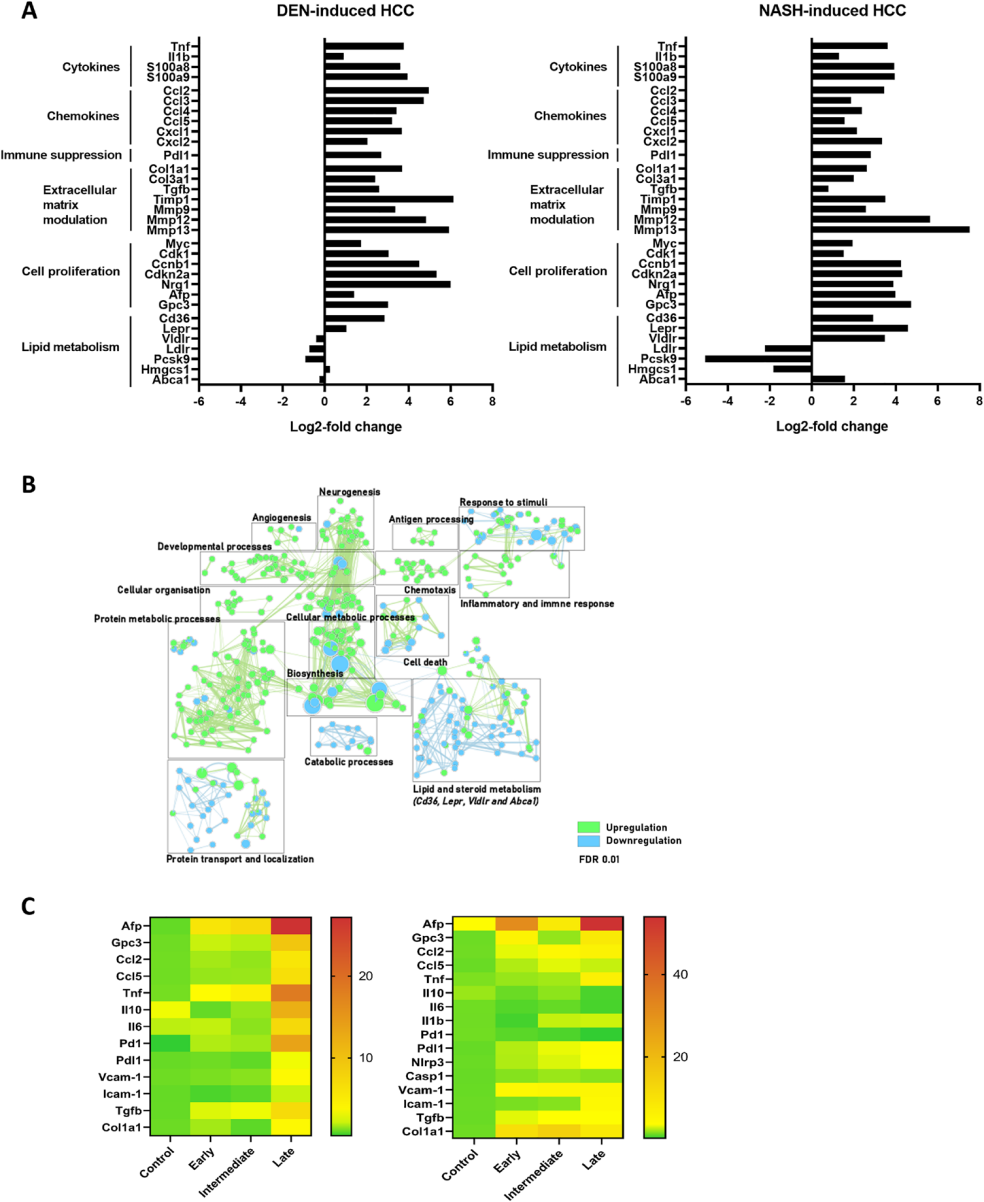
(**A**) Differential gene expression in full liver tissue in DEN-induced HCC and NASH-induced HCC, comparing control (*n* = 4) and late stage (*n* = 4) HCC development. (**B**) Gene enrichment mapping of significantly (FDR 0.01) up- or downregulated (green and blue, respectively) GO pathways comparing the DEN- and NASH-HCC model at end-stage HCC development (*n* = 4). (**C**) Heatmap of RT-qPCR gene expression in full liver tissue (mean power value, data log transformed) in controls, early, intermediate and late stage HCC development. DEN-model (left): control (*n* = 7), early (*n* = 9), intermediate (*n* = 10) and end-stage (*n* = 10). NASH-model (right): control (*n* = 5), early (*n* = 10), intermediate (*n* = 12) and end-stage (*n* = 6).

In both the DEN- and NASH-induced HCC model, there was a strong activation of inflammation-related pathways, including an upregulation of pro-inflammatory cytokines (including Tnf, Il1b, S100a8 and S100a9) and chemokines (e.g., Ccl2, Ccl3, Ccl4, Ccl5, Cxcl1 and Cxcl2) (Figure 2A and 2B; Figure 3A). These genes are key factors in the immune response to stimuli, leucocyte differentiation and activation chemotaxis of immune cells to areas of tissue injury. On the other hand, Pdl1, which regulates the immune response and is considered a marker for immune suppression, was also upregulated, indicating a complex balance between pro- and anti-inflammatory factors. Genes involved in the regulation of fibrosis, such as various collagens and matrix metalloproteinases, were also upregulated.

As expected, the progression to HCC was characterized by an induced expression of genes involved in cell cycle regulation and proliferation (Myc, Cdk1, Ccnb1, Cdkn2a, Nrg1). The HCC markers Afp and Gpc3 were upregulated as well (Figure 2A and 2B; Figure 3A).

When evaluating differentially expressed genes between liver tissue from the end-stage DEN- and NASH-HCC, 942 and 701 genes were up- and downregulated, respectively (adjusted *p*-value < 0.05). Notably, gene enrichment mapping showed that in the NASH-HCC model, mainly genes involved in lipid and steroid metabolism were differentially regulated. This included both the up- and downregulation of key genes such as the LDL (Ldlr), VLDL (Vldlr) and leptin (Lepr) receptors, the Ldlr-associated protein Pcsk9 and the fatty acid transporter CD36 (Figure 3A and 3B).

Importantly, the expression of genes coding for cytokines and chemokines, and genes involved in matrix remodeling and cell proliferation gradually increased over time from control mice to intermediate to end-stage HCC. (Supplementary Figure 1) These findings were confirmed by RT-qPCR analysis of Tnf, Ccl2, Ccl5, Pdl1, Col1a1, Tfgb, Afp and Gpc3 (Figure 3C).

### Hepatic Mφ subsets display distinct features in HCC progression

**Figure 4:**
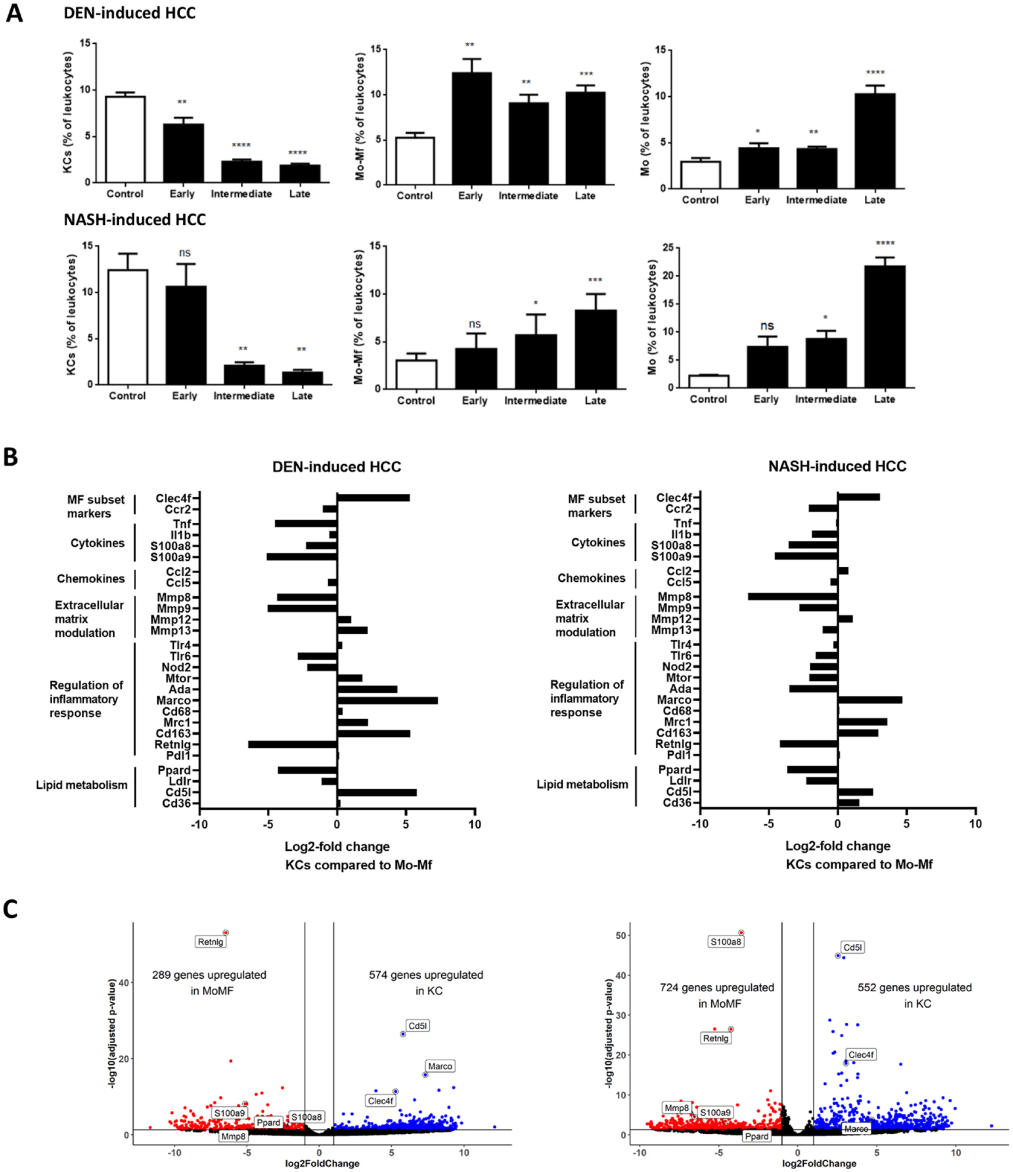
(**A**) Mφ subtypes as percentage of leukocytes (CD45+ cells) during HCC. DEN-model: control (*n* = 7), early (*n* = 9), intermediate (*n* = 10) and end-stage (*n* = 10). NASH-model: control (*n* = 5), early (*n* = 10), intermediate (*n* = 12) and end-stage (*n* = 6). Variables are presented as mean ± SEM. Multiple comparisons analysis was performed using ANOVA. ^*^ *P* < 0.05, ^**^ *P* < 0.01, ^***^ *P* < 0.001 compared to control. (**B**) Differential gene expression in KCs compared to Mo-Mφ at end-stage HCC development in DEN-induced HCC (*n* = 4) and NASH-induced HCC model (*n* = 4) (**C**) Volcano plots for KCs compared to Mo-Mφ at end-stage HCC development in both models.

To characterize the different hepatic Mφ populations during HCC development, flow cytometric analysis of KCs, Mo and Mo-Mφ populations at different stages of HCC development was performed. KCs were depleted at onset of disease in the DEN model, and further decreased at intermediate and end-stage HCC in both models. The proportion of infiltrating Mo-Mφ was already markedly increased at early stage DEN-HCC and remained high at the intermediate and end-stage time points. In the NASH model, Mo-Mφ gradually increased from early to end-stage HCC. From the onset of HCC development, the percentage of Mo steadily increased at early and intermediate stage, with a marked increase at end-stage HCC (Figure 4A).

We next performed RNA sequencing analysis on FACS-isolated cells to compare the transcriptional profile of KC, Mo and Mo-Mφ during HCC development. DEN- and NASH-induced HCC resulted in differential gene expression (adjusted *p*-value < 0.05) between the 3 cell types. Corroborating our gating strategy, characteristic Mφ identification markers such as Clec4f and Cd5l for KCs, and CCR2 and Retnlg for Mo-Mφ and Mo, differed significantly between the Mφ subsets (Figure 4B and 4C and Supplementary Figure 2A and 2B).

The functional roles of KCs versus Mo-Mφ were conserved between the two HCC models. Modulation of the extracellular matrix and stimulation of inflammation were mostly, but not exclusively, mediated by Mo-Mφ. This differential expression was also present for Tlr6 and Nod2, two pattern recognition receptors involved in pro-inflammatory cytokine production and activation of the innate immune response [49, 50]. On the other hand, chemotaxis was relatively equally mediated by all cell types (Figure 4B and Supplementary Figure 1A and 1B). There was also no difference in expression of the M1-like marker Cd68 or the immune exhaustion marker Pdl1 between the different cell types, whereas the immune-suppressive (or M2-like markers) Mrc1 and Cd163 were high in KCs and Mo-Mφ compared to Mo [51, 52]. The immunomodulatory scavenger receptor Marco was upregulated in KCs compared to Mo- Mφ and Mo. Interestingly, metabolic regulators of immune activation such as mTOR and Ada were higher in KCs in the DEN-induced HCC model, yet higher in Mo-Mφ in NASH-induced HCC, indicating highly context-dependent roles of these genes in the integration of metabolism and inflammation. Ppard expression was higher in Mo-Mφ (Figure 4B), which is in accordance with the postulated immunomodulatory role for PPARδ in infiltrating macrophages [53].

In conclusion, the phenotype of liver Mφ subsets is complex and does not neatly fit into the simplified pro-inflammatory (M1) or anti-inflammatory/pro-tumorigenic (M2) spectrum of macrophage polarization.

### Context-dependent hepatic Mφ activation in HCC

**Figure 5:**
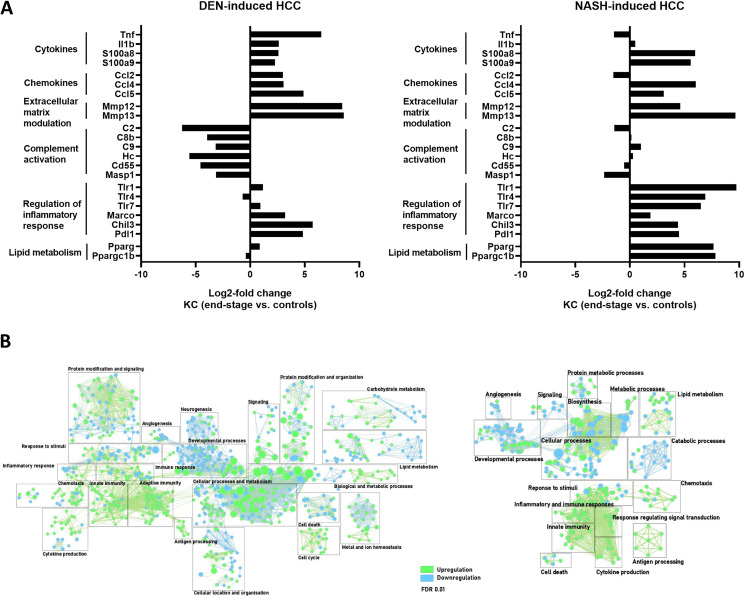
(**A**) Differential gene expression in KC comparing end-stage and controls in DEN- (*n* = 4) and NASH-induced HCC (*n* = 4). (**B**) Gene enrichment mapping of significantly (FDR 0.01) up- or downregulated (green and blue, respectively) GO pathways comparing KC in end-stage and controls in DEN- (*n* = 4) and NASH-induced HCC (*n* = 4).

**Figure 6:**
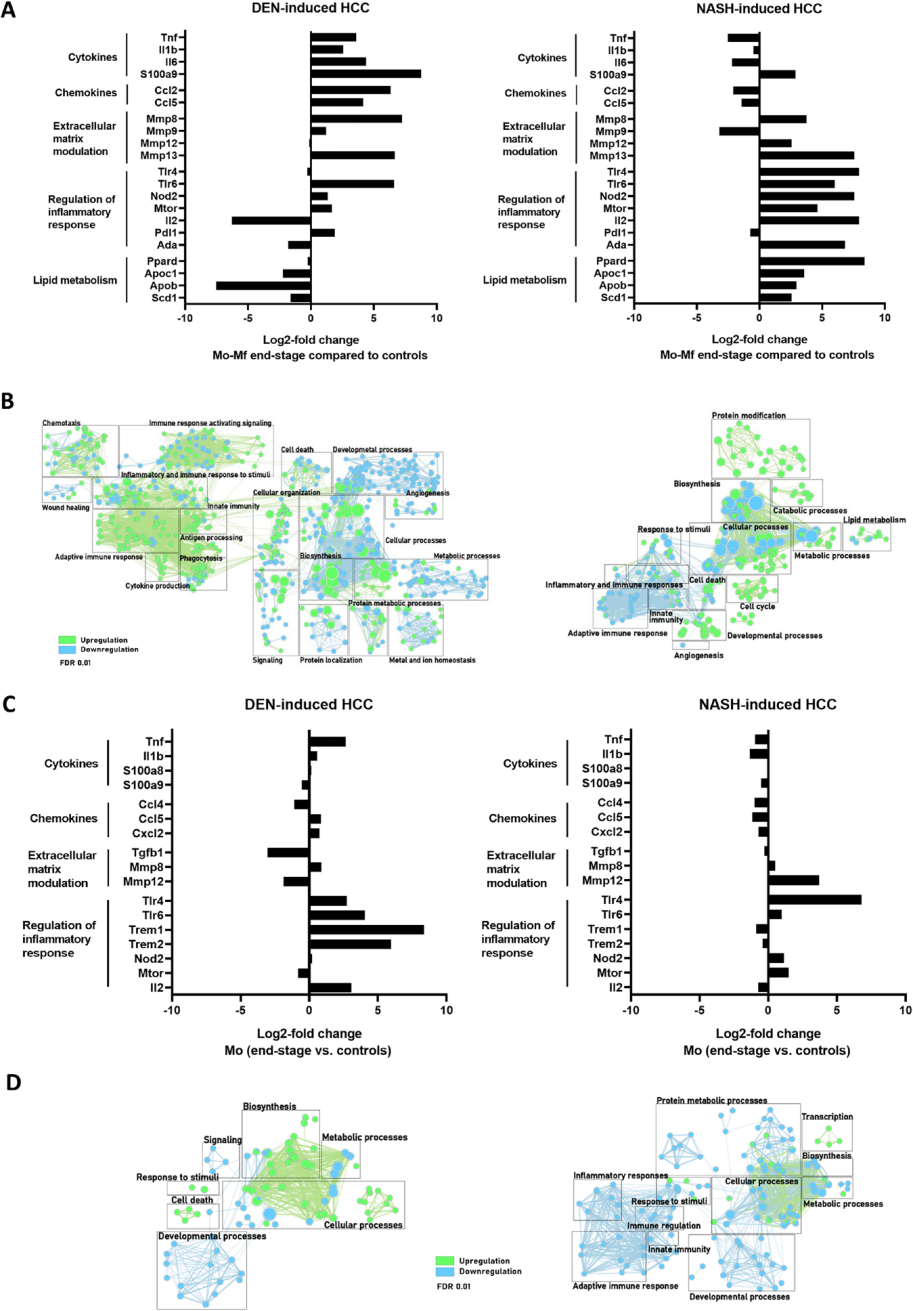
(**A**, **C**) Differential gene expression in Mo-Mφ (A) and Mo (C) comparing end-stage and controls in DEN- (*n* = 4) and NASH-induced HCC (*n* = 4). (**B**, **D**) Gene enrichment mapping of significantly (FDR 0.01) up- or downregulated (green and blue, respectively) GO pathways comparing Mo-Mφ (B) and Mo (D) in end-stage and controls in DEN- (*n* = 4) and NASH-induced HCC (*n* = 4).

We then analyzed the longitudinal changes in gene expression during the development of HCC in these two models. DEN and NASH-induced HCC resulted in differential gene expressions at end-stage HCC in all analysed liver Mφ subsets and this was most pronounced for KCs and Mo-Mφ (Figure 5; Figure 6A and 6B), while less changes were observed in Mo (Figure 6C and 6D), in part because after infiltration, these Mo differentatiate into Mo-Mφ.

In both DEN-and NASH-induced HCC, pathways related to innate immunity were upregulated in KCs, as evidenced by a higher expression of genes coding for pro-inflammatory cyto- and chemokines such as S100a8, S100a9, Ccl4 and CCl5 (Figure 5A and 5B). Simultaneously, the immune-supressive genes Marco, Chil3, Pdl1 were also upregulated at end-stage disease. Notably, the complement activation pathway was significantly downregulated in DEN-induced HCC but unaffected in NASH-HCC. In this regard, a reduced expression for the complement factor C2 for instance was shown to correlate with a worse prognosis in HCC [54].

Stark differences between the models were also observed in Mo-Mφ gene expression patterns (Figure 6A and 6B). Specifically, inflammatory pathways and cytokines were upregulated in DEN-HCC, which was expected given the inflammatory nature of the tumor micro-environment. Conversely, Mo-Mφ in the NASH-HCC model displayed a distinct phenotype, characterized by a mixed pro-inflammatory expression profile and the upregulation of immunomodulatory (Tlr4, Tlr6, Mtor, Ada) and lipid metabolic (Apoc2, Apob, Scd1) genes [55].

The profibrogenic polarization of hepatic KCs and Mo-Mφ in chronic liver injury was confirmed by the elevated expression of genes involved in extracellular matrix modulation and fibrosis (Figures 5 and 6).

Interestingly, in KC, the expression of Pparg and its cofactor Ppargc1b was increased, whereas in Mo-Mφ, it was the PPARδ isoform which was upregulated in the context of NASH-HCC. These nuclear receptors not only regulate fatty acid storage and glucose metabolism, but they also play a role in macrophage-mediated inflammation in experimental NAFLD [53].

Taken together, KCs and Mo- Mφ have a mixed pro-inflammatory and immune-suppressive phenotype, with an emphasis on immunoregulatory and lipid metabolism expression profiles in the NASH-HCC model (Figures 5 and 6).

## DISCUSSION

Hepatic macrophages are key players in tumor initiation and progression. They sustain a tumor-promoting pro-inflammatory microenvironment, and at the same time respond to tumor- and stromal cell-derived signals to actively facilitate HCC progression. As such, the abundantly present TAMs in the TME represent attractive targets for novel therapies, which is highly relevant given that few therapeutic options are available for non-resectable HCC. Although considerable progress has been made in understanding the role of different Mφ populations in HCC, numerous questions remain. A phenotypical characterization of specific macrophage subsets would provide a more in-depth understanding of cellular heterogeneity in the inflamed environment of a fibrotic liver. In this study, we investigated the inflammatory TME and isolated Mφ populations during HCC development in 2 different, frequently used, experimental HCC mouse models. DEN is the most extensively used genotoxic agent for chemically-induced HCC. Since NAFLD is projected to become the leading cause of chronic liver injury, and since NAFLD is associated with an increased risk for HCC development, there is a need for more research on dietary models of HCC. We here contribute to the current knowledge in the field by 1) unravelling the transcriptional profile of isolated hepatic Mφ subsets, showing clearly distinct phenotypical characteristics, with mixed inflammatory and pro-tumoral polarized expression profiles, which shift during HCC development; and 2) deciphering the functional differences in Mφ populations in HCC, which is heavily influenced by the microenvironment and underlying disease etiology, such as the importance of metabolic immune regulators in NASH-HCC.

In both experimental models, tumoral lesions developed over time. Characterization of the inflammatory micro-environment by RNA sequencing analysis of liver tissue revealed gradual transcriptomic changes during tumor development, including the induction of inflammatory, immunoregulatory, profibrotic and proliferative pathways. The variety of induced growth factors, cytokines and matrix modulating proteins has been reported to contribute to HCC tumor initiation, progression and invasiveness [56–61]. As expected, in the NASH-model, genes involved in lipid, fatty acid and steroid metabolism were upregulated compared to the DEN-model.

KC depletion following an acute insult is well-established, while the composition of liver Mφ during hepatic carcinogenesis in general, is less clear [62–69]. Our recent findings, which rely on the KC-specific marker Tim4 (compared to other hepatic macrophage populations) enabling the distinction between KCs and Mo-Mφ [33, 37, 38], show that KCs are also depleted and Mo and Mo-Mφ rise in experimental HCC [41]. In the tumoral micro-environment, chemokines secreted by malignant and stromal cells and activated KCs recruit bone marrow-derived Mo. Given the Mφ dynamics during tumor development, it is assumed that the majority of TAMs are derived from these infiltrating Mo [11, 20, 70]. However, the relative contribution of KCs, Mo-Mφ and Mo to the TAM population during different stages of HCC development is still debated. To address this issue, we characterized the functional phenotype of the Mφ subsets and first compared the transcriptional profile of the different Mφ subsets and confirmed previous reports on the phenotypical difference between liver KCs, Mo-Mφ and Mo at steady state and in NASH [35, 55] and NASH-HCC [71]. Characteristic differentiation markers like Clec4f for KCs and CCR2 for Mo-Mφ and Mo are strongly divergent between the Mφ subsets. Importantly, we observed that pro-tumoral and immune-suppressive markers, but also pro-inflammatory genes, are expressed by both KCs and Mo-Mφ. Tumor cells utilize this pro-inflammatory environment with the aim of tissue re-modelling, angiogenesis, and stimulation of migration and invasion. These data confirm that at least some Mo that differentiate into Mo-Mφ acquire a pro-tumoral phenotype, but also show that KCs are part of the TAM population and enhance tumor progression [17, 58, 72–74], although they were initially thought to be only involved in anti-tumor immunity. Indeed, our data suggest that Mφ activation patterns are complex, and therefore not reducible to a simple dichotomous inflammatory (M1-like) or anti-inflammatory (M2-like) polarization state, and that both resident and infiltrating liver Mφ contribute to tumor progression.

We then evaluated transcriptional changes within each cell subset over time in both models. KCs and Mo-Mφ underwent the most pronounced phenotypical changes. Pathways involved in immune cell activation, regulation of the immune system and immune response as well as fibrogenesis were gradually upregulated in all cell types. Nevertheless, the most striking findings were the major differences in pathways and specific genes between the two disease models. The upregulation of anti-inflammatory and/or immunomodulatory genes was more evident in KCs and Mo-Mφ in the NASH-HCC model. Further research is needed to elucidate the underlying mechanisms, yet we propose this might be linked to an activation of lipid metabolic pathways and the subsequent accumulation of immunometabolites [75]. The activation of the PPARγ and δ nuclear receptors in KCs and Mo-Mφ, respectively, is one potential factor involved [53].

The optimal approach to target specific Mφ subsets as therapy in HCC will need to be further explored. Potential strategies could include decreasing the population of TAMs by blocking the recruitment of bone-marrow derived Mo, but also functionally reprogramming of TAMs to an anti-tumoral phenotype. The latter approach might be necessary since also resident KCs have a clear pro-tumoral phenotype. Our description of the time-dependent phenotypical changes during tumor initiation and progression can guide future experiments in timing of preventive or therapeutic interventions. The next challenge is to translate this to human disease. At present, comparative studies on human versus mouse liver Mφ subsets are limited, although many key aspects of KCs and Mo-Mφ activation, recruitment signals, and metabolic activities appear to be conserved [76, 77].

To conclude, HCC is an inflammation driven tumor, in which hepatic macrophage populations play a central role. KCs, Mo and Mo-Mφ have divergent phenotypes that change with disease progression and these cell populations contribute to the population of TAMs in HCC. The knowledge that KCs also express typical TAM markers further contributes to the understanding of hepatocarcinogenesis in two different frequently used experimental mouse HCC models.

## MATERIALS AND METHODS

### Animal experiments

Five-week-old male SV129 wild-type mice were purchased from Janvier Labs (Le Genest-Saint-Isle, France). C57BL/6J mice (Janvier Labs) were bred in the animal facility. The animals were housed at room temperature (20–24°C) and constant humidity (45–65%) in a 12-hour controlled dark/light cycle with unrestricted access to food and water at the animal facility of the Faculty of Medicine and Health Sciences, Ghent University, Belgium. The welfare of all animals was daily evaluated and the mice were weekly weighted, during the entire duration of the experiment. All animal experiments were reviewed and approved by the Animal Ethics Committee of the Faculty for Medicine and Health Sciences, University Ghent (ECD16/64).

### Animal models

#### Diethylnitrosamine (DEN)-induced HCC model

Wild-type male 129/Sv mice were injected weekly intraperitoneally with 35 mg/kg DEN (Sigma-Aldrich, Diegem, Belgium) or 0.9% NaCL for 30 weeks, starting at 5 weeks of age. At week 5 (*n* = 4), 10 (*n* = 5), 15 (*n* = 5), 20 (*n* = 5), 25 (*n* = 5) and 30 (*n* = 5) of DEN-injections, mice were sacrificed. Samples of week 5 and 10 were grouped as ‘early stage’, week 15 and 20 as ‘intermediate stage’ and week 25 and 30 as ‘late stage’ disease. Wild-type male 129/Sv mice with saline-injection weekly were used as controls (*n* = 7) and sacrificed at week 25. All groups were fed a normal chow diet (Pavan Service-Carfil, Oud-Turnhout, Belgium).

### Non-alcoholic steatohepatitis (NASH)-induced HCC model

Wild-type C57BL/6J mice were injected subcutaneously with 200 μg STZ, Sigma, Overijse, Belgium) dissolved in Hanks’ balanced salt solution (Invitrogen, Ghent, Belgium) 2 days after birth to destroy the pancreatic β-cells. Male mice were fed a high-fat, high-sucrose, high-cholesterol diet (Western diet, Ssniff, Uden, The Netherlands, TD.08811 + 1% added cholesterol) from 4 weeks to 16 weeks of age. At week 4 (*n* = 5), 6 (*n* = 5), 8 (*n* = 6), 12 (*n* = 6), and 16 (*n* = 6) of age mice were sacrificed. Samples of week 4 and 6 were grouped as ‘early stage’, week 8 and 12 as ‘intermediate stage’ and week 16 as ‘late stage’ disease. Control wild-type male C57BL/6J mice did not receive STZ injections and were fed a normal chow diet (Pavan Service-Carfil) (*n* = 5) and sacrificed at week 16.

### Sample collection

For tissue sampling, C57BL/6J mice were fasted overnight. At the day of sacrifice, mice were weighted, and anesthetized by intraperitoneal injection of a ketamine (100 mg/kg)–xylazine (10 mg/kg) solution. The mice were euthanized via cervical dislocation prior to dissection. Retro-orbital blood samples were taken and serum was stored at −80°C. Spleen and liver were isolated and weighed. Macroscopically visible hepatic tumor nodules were counted and subdivided according to size. The right liver lobe was flushed with PBS, isolated and placed in a graduated falcon tube containing 2 ml of RPMI. The left liver lobe was divided for RNA analysis and histology. The parts of the liver for RNA analysis were collected in RNA-later (Invitrogen, Thermo Fisher Scientific, Ghent, Belgium), snap-frozen in liquid nitrogen and stored at –80°C until further analysis. Tissue sections for histological examination were collected and fixed in 4% formaldehyde solution (Klinipath, VWR, Leuven, Belgium) and paraffin-embedded for Sirius Red and haematoxylin and eosin (H&E) stainings.

### Evaluation of glucose homeostasis

STZ-induced diabetes development was evaluated at sacrification, C57BL/6J mice were fasted overnight and fasting blood glucose levels were measured in tail vein blood samples with a glucometer (Bayer Contour Next, Ascensia Diabetes Care, Basel, Switzerland). An oral glucose tolerance test was performed. Mice were given 2 g/kg glucose (Sigma-Aldrich) in 0.9% NaCl solution by oral gavage. Blood glucose levels were measured at 0, 30, 60, 90 and 120 min after glucose administration.

### Histology

Paraffin-embedded liver tissue was sectioned (Leica RM2145, Leica Biosystems, Diegem, Belgium), dried and rehydrated by serial immersion in xylene (Klinipath) and ethanol (VWR, Leuven, Belgium). H&E staining was performed by incubation in Haematoxylin for 3 min (Mayer, SigmaAldrich), H2O for 15 min, 96% EtOH for 1 min, and 1% Eosin and 0.5% glacial acetic acid (VWR, Leuven Belgium) in 90% EtOH for 5 min. Histology slides for Sirius Red staining were incubated for 30 min in 1% Sirius Red (Klinipath) and 1,3% picric acid (Sigma-Aldrich) in H2O, followed by a 10 min incubation in H2O. All sections were then dehydrated by serial immersion in ethanol and xylene. All histological slides were blinded and scored by two independent researchers.

NASH progression was evaluated by H&E staining of liver tissue. The NAFLD activity score (NAS) was used to describe NASH severity. The scoring includes measurement of steatosis grade (0 - < 5% steatosis, 1 – 5–33% steatosis, 2 - 33–66% steatosis, 3 - > 66% steatosis), hepatocyte ballooning (0 - none, 1- few ballooning cells, 2 - many cells/prominent ballooning) and inflammation (0 - no foci, 1 - < 2 foci, 2 – 2–4 foci, 3 - > 4 foci). Fibrosis levels were evaluated on Sirius Red stained liver sections using the Metavir score and NAFLD fibrosis score in the DEN and NASH-HCC models, respectively [78].

### Liver single-cell suspensions

The right liver lobe was flushed with PBS, isolated and placed in a graduated falcon tube containing 2 ml of RPMI. After this the liver was dissociated using scissors followed by gentleMACS (Miltenyi Biotec, Bergisch Gladbach, Germany) and digested for 20 minutes in 1 mg/ml Collagenase A (Roche, Sigma-Aldrich) and 10 U/ml DNAse (Roche, Sigma-Aldrich) in RPMI (Gibco, Thermo Fisher Scientific, Ghent, Belgium) under frequent agitation in a warm water bath at 37°C, and further dissociated using gentleMACS. Further steps were executed on ice. RPMI was added to adjust the volume of both cell suspensions to 50 mL, after which they were filtered through a 100 μm cell strainer (Greiner Bio-one, Vilvoorde, Belgium), and centrifuged for 5 min at 1311 rpm and 4°C. The supernatant was discarded and cells were incubated with red blood cell lysis buffer (155 mM NH4Cl, 10 mM KHCO3, 0.1 mMEDTA, pH 7.2) for 45 s. Ice cold PBS was added to adjust the volume to 50 mL followed by centrifugation (5 min, 1311 rpm, 4°C). The cell suspension was filtered through a 40 μm cell strainer (Greiner Bio-one), transferred to a V-bottom 96-well plate and washed with 2% heat inactivated fetal bovine serum (FBS, Gibco, Thermo Fisher Scientific, Ghent, Belgium) and 2 mM EDTA (Sigma-Aldrich) in PBS (FACS buffer). After antigen blockade with rat anti-mouse CD16/32 antibody Fc block (BD Biosciences, Erembodegem, Belgium), the cells were stained with CD45/APC-Cy7, Ly6C/V450, CD11b/PE-Cy7, Ly6G/PerCP-Cy5.5, Tim4/PE (BD Biosciences) and F4/80/FITC (Thermo Scientific, Merelbeke, Belgium) for 20 min at 4°C in the dark.

### Flow cytometry and sorting

After staining, the cells were analyzed with a FACS Aria III cell sorter (BD Biosciences) and FLowJo software (FlowJo LLC, BD Bioscences). A gating strategy was employed as previously described [41]. Briefly, cells were gated as single cells and subsequently as CD45+ Ly6G- Ly6C+ CD11b+ Tim4- monocytes (Mo), CD45+ F4/80int Ly6G- Ly6C- CD11b+ Tim4- monocyte-derived macrophages (Mo-Mφ) and CD45+ F4/80+ Ly6G- Ly6C- CD11bInt Tim4+ Kupffer cells (KCs) [33, 37, 38]. These cells were sorted and isolated cells were collected in RLT buffer (Qiagen, Stokach, Germany) supplemented with 1% β-mercaptoethanol, mixed by vortexing, frozen on dry ice, and stored by −80°C until analysis.

### RNA extraction and quantitative RT-qPCR

RNA was extracted from 20 mg mouse liver tissue (surrounding liver and tumors) preserved in RNAlater (Invitrogen) using the Qiagen RNeasy Mini Kit (Qiagen Benelux, Venlo, The Netherlands). mRNA from isolated cell populations was extracted using the RNeasy micro kit (Qiagen), according to the manufacturer’s protocol. The RNA quality was evaluated by spectrophotometry, calculating the A260/A280 ratio. cDNA synthesis was performed starting from 1 μg RNA, using the SensiFAST cDNA synthesis kit (Bioline, London, UK). cDNA was added to a 384-well plate with specific primers (Biolegio, Nijmegen, The Netherlands) and Sensimix SYBR No-ROX Mastermix (Bioline). Samples were run and analyzed on the Lightcycler 480 II (Roche). Measurements were performed in duplicate and Cq values were calculated with the second derivative maximum method. Average Cq values were normalized to the Cq of stable housekeeping genes, according to analysis in GeNorm (Biogazelle, Ghent, Belgium).

### RNA sequencing

Liver tissue was sequenced from control mice (*n* = 4), mice at week 15 (defined as intermediate stage, *n* = 4) and week 30 (end-stage, *n* = 4) in the DEN-model. In the NASH-induced model control mice (*n* = 4), mice at 8 weeks of age (intermediate stage, *n* = 4) and 16 weeks of age (end-stage, *n* = 4) were used. For the different cell populations, *n* = 4 for all groups (except Mo in NASH-induced HCC, *n* = 3). After RNA extraction, the concentration and quality of the total extracted RNA were checked by using the ‘Quant-it ribogreen RNA assay’ (Life Technologies, Grand Island, NY, USA) and the RNA lab chip (Perkin Elmer, Waltham, MA, USA), respectively. Subsequently, 500 ng of RNA for the liver samples and 2.26 ng of RNA for the remaining samples were used to perform an Illumina sequencing library preparation using the QuantSeq 3′ mRNA-Seq Library Prep Kit (Lexogen, Vienna, Austria) according to the manufacturer’s protocol. During library preparation, 14 PCR cycles for the liver samples and 21 PCR cycles were used. Libraries were quantified by qPCR, according to Illumina’s protocol ‘Sequencing Library qPCR Quantification protocol guide’, version February 2011. A High Sensitivity DNA chip (Agilent Technologies, Santa Clara, CA, USA) was used to control the library’s size distribution and quality. Sequencing was performed on a high throughput Illumina NextSeq 500 flow cell generating 75 bp single reads. Per sample, on average 9.2 ± 4.9 million reads were generated. First, these reads were trimmed using Trimmomatic software version 0.39 to remove the “QuantSEQ FWD” adaptor sequence and the remove low quality bases. The trimmed reads were mapped against the Mus musculus GRCm38.98 reference genome STAR software version 2.7.3a. The RSEM software, version 1.3.3, was used to generate the count tables. Differential gene expression analysis between groups of samples was performed using DESeq2, version 1.26.0. Genes having a false discovery rate lower than or equal to 0.01 and a log fold change large or equal to two were considered differentially expressed. Differentially expressed genes were used for gene set enrichment analyses (GO biological process) using Cytoscape and BiNGO plug-in.

### Statistical analysis

Statistical analysis was performed using SPSS statistics 24 software (IBM analytics, Brussels, Belgium) and graphical representations were made using Graphpad Prism (Graphpad Software Inc., San Diego, CA, USA). Multiple-group comparisons were performed using analysis of variance (ANOVA). A value of *P* < 0.05 was considered statistically significant (^*^ *P* < 0.05, ^**^ *P* < 0.01, ^***^ *P* < 0.001). Continuous variables are presented as mean ± SEM, gene expression data as the geometric mean ± 95% confidence interval.

## SUPPLEMENTARY MATERIALS



## Abbreviations

Afp: Alpha-fetoprotein
DEN: Diethylnitrosamine
HCC: Hepatocellular carcinoma
FACS: Fluorescence-Activated Cell Sorter
Gpc3: Glypican 3
KCs: Kupffer cells
Mφ: Macrophage
Mo: Monocytes
Mo-Mφ: Monocyte-derived Macrophages
NAFLD: Non-Alcoholic Fatty Liver Disease
NASH: Non-Alcoholic Steatohepatitis
NS: Non-Significant
STZ: Streptozotocin
TAMs: Tumor Associated Macrophages
TME: Tumor Microenvironment

## References

[1] European Association for the Study of the Liver. Electronic address: easloffice@easloffice.eu; European Association for the Study of the Liver. EASL Clinical Practice Guidelines: Management of hepatocellular carcinoma. J Hepatol. 2018; 69:182–236. 10.1016/j.jhep.2018.03.019. Erratum in: J Hepatol. 2019; 70:817. . 29628281

[2] Tolba R , Kraus T , Liedtke C , Schwarz M , Weiskirchen R . Diethylnitrosamine (DEN)-induced carcinogenic liver injury in mice. Lab Anim. 2015; 49:59–69. 10.1177/0023677215570086. 25835739

[3] Heindryckx F , Colle I , Van Vlierberghe H . Experimental mouse models for hepatocellular carcinoma research. Int J Exp Pathol. 2009; 90:367–86. 10.1111/j.1365-2613.2009.00656.x. 19659896 PMC2741148

[4] Fausto N , Campbell JS . Mouse models of hepatocellular carcinoma. Semin Liver Dis. 2010; 30:87–98. 10.1055/s-0030-1247135. 20175036

[5] Lee JS , Chu IS , Mikaelyan A , Calvisi DF , Heo J , Reddy JK , Thorgeirsson SS . Application of comparative functional genomics to identify best-fit mouse models to study human cancer. Nat Genet. 2004; 36:1306–11. 10.1038/ng1481. 15565109

[6] Friemel J , Frick L , Unger K , Egger M , Parrotta R , Boge YT , Adili A , Karin M , Luedde T , Heikenwalder M , Weber A . Characterization of HCC Mouse Models: Towards an Etiology-Oriented Subtyping Approach. Mol Cancer Res. 2019; 17:1493–1502. 10.1158/1541-7786.MCR-18-1045. 30967480

[7] Takakura K , Koido S , Fujii M , Hashiguchi T , Shibazaki Y , Yoneyama H , Katagi H , Kajihara M , Misawa T , Homma S , Ohkusa T , Tajiri H . Characterization of non-alcoholic steatohepatitis-derived hepatocellular carcinoma as a human stratification model in mice. Anticancer Res. 2014; 34:4849–55. 25202066

[8] Hernandez-Gea V , Toffanin S , Friedman SL , Llovet JM . Role of the microenvironment in the pathogenesis and treatment of hepatocellular carcinoma. Gastroenterology. 2013; 144:512–27. 10.1053/j.gastro.2013.01.002. 23313965 PMC3578068

[9] Tahmasebi Birgani M , Carloni V . Tumor Microenvironment, a Paradigm in Hepatocellular Carcinoma Progression and Therapy. Int J Mol Sci. 2017; 18:405. 10.3390/ijms18020405. 28216578 PMC5343939

[10] Heindryckx F , Gerwins P . Targeting the tumor stroma in hepatocellular carcinoma. World J Hepatol. 2015; 7:165–76. 10.4254/wjh.v7.i2.165. 25729472 PMC4342599

[11] Rani B , Cao Y , Malfettone A , Tomuleasa C , Fabregat I , Giannelli G . Role of the tissue microenvironment as a therapeutic target in hepatocellular carcinoma. World J Gastroenterol. 2014; 20:4128–40. 10.3748/wjg.v20.i15.4128. 24764651 PMC3989949

[12] Sachdeva M , Chawla YK , Arora SK . Immunology of hepatocellular carcinoma. World J Hepatol. 2015; 7:2080–90. 10.4254/wjh.v7.i17.2080. 26301050 PMC4539401

[13] Chew V , Tow C , Teo M , Wong HL , Chan J , Gehring A , Loh M , Bolze A , Quek R , Lee VK , Lee KH , Abastado JP , Toh HC , Nardin A . Inflammatory tumour microenvironment is associated with superior survival in hepatocellular carcinoma patients. J Hepatol. 2010; 52:370–9. 10.1016/j.jhep.2009.07.013. 19720422

[14] Balkwill F , Mantovani A . Inflammation and cancer: back to Virchow? Lancet. 2001; 357:539–45. 10.1016/S0140-6736(00)04046-0. 11229684

[15] Aggarwal BB , Shishodia S , Sandur SK , Pandey MK , Sethi G . Inflammation and cancer: how hot is the link? Biochem Pharmacol. 2006; 72:1605–21. 10.1016/j.bcp.2006.06.029. 16889756

[16] Fang M , Yuan J , Chen M , Sun Z , Liu L , Cheng G , Ying H , Yang S , Chen M . The heterogenic tumor microenvironment of hepatocellular carcinoma and prognostic analysis based on tumor neo-vessels, macrophages and alpha-SMA. Oncol Lett. 2018; 15:4805–4812. 10.3892/ol.2018.7946. 29552120 PMC5840703

[17] Dong P , Ma L , Liu L , Zhao G , Zhang S , Dong L , Xue R , Chen S . CD86(+)/CD206(+), Diametrically Polarized Tumor-Associated Macrophages, Predict Hepatocellular Carcinoma Patient Prognosis. Int J Mol Sci. 2016; 17:320. 10.3390/ijms17030320. 26938527 PMC4813183

[18] Wu K , Kryczek I , Chen L , Zou W , Welling TH . Kupffer cell suppression of CD8+ T cells in human hepatocellular carcinoma is mediated by B7-H1/programmed death-1 interactions. Cancer Res. 2009; 69:8067–75. 10.1158/0008-5472.CAN-09-0901. 19826049 PMC4397483

[19] Yeung OW , Lo CM , Ling CC , Qi X , Geng W , Li CX , Ng KT , Forbes SJ , Guan XY , Poon RT , Fan ST , Man K . Alternatively activated (M2) macrophages promote tumour growth and invasiveness in hepatocellular carcinoma. J Hepatol. 2015; 62:607–16. 10.1016/j.jhep.2014.10.029. 25450711

[20] Shirabe K , Mano Y , Muto J , Matono R , Motomura T , Toshima T , Takeishi K , Uchiyama H , Yoshizumi T , Taketomi A , Morita M , Tsujitani S , Sakaguchi Y , Maehara Y . Role of tumor-associated macrophages in the progression of hepatocellular carcinoma. Surg Today. 2012; 42:1–7. 10.1007/s00595-011-0058-8. 22116397

[21] Capece D , Fischietti M , Verzella D , Gaggiano A , Cicciarelli G , Tessitore A , Zazzeroni F , Alesse E . The inflammatory microenvironment in hepatocellular carcinoma: a pivotal role for tumor-associated macrophages. Biomed Res Int. 2013; 2013:187204. 10.1155/2013/187204. 23533994 PMC3591180

[22] Tian Z , Hou X , Liu W , Han Z , Wei L . Macrophages and hepatocellular carcinoma. Cell Biosci. 2019; 9:79. 10.1186/s13578-019-0342-7. 31572568 PMC6761725

[23] Dong N , Shi X , Wang S , Gao Y , Kuang Z , Xie Q , Li Y , Deng H , Wu Y , Li M , Li JL . M2 macrophages mediate sorafenib resistance by secreting HGF in a feed-forward manner in hepatocellular carcinoma. Br J Cancer. 2019; 121:22–33. 10.1038/s41416-019-0482-x. 31130723 PMC6738111

[24] Yao RR , Li JH , Zhang R , Chen RX , Wang YH . M2-polarized tumor-associated macrophages facilitated migration and epithelial-mesenchymal transition of HCC cells via the TLR4/STAT3 signaling pathway. World J Surg Oncol. 2018; 16:9. 10.1186/s12957-018-1312-y. 29338742 PMC5771014

[25] Sawa-Wejksza K , Kandefer-Szerszen M . Tumor-Associated Macrophages as Target for Antitumor Therapy. Arch Immunol Ther Exp (Warsz). 2018; 66:97–111. 10.1007/s00005-017-0480-8. 28660349 PMC5851686

[26] Mantovani A , Marchesi F , Malesci A , Laghi L , Allavena P . Tumour-associated macrophages as treatment targets in oncology. Nat Rev Clin Oncol. 2017; 14:399–416. 10.1038/nrclinonc.2016.217. 28117416 PMC5480600

[27] Petty AJ , Yang Y . Tumor-associated macrophages: implications in cancer immunotherapy. Immunotherapy. 2017; 9:289–302. 10.2217/imt-2016-0135. 28231720 PMC5619052

[28] Mantovani A , Locati M . Tumor-associated macrophages as a paradigm of macrophage plasticity, diversity, and polarization: lessons and open questions. Arterioscler Thromb Vasc Biol. 2013; 33:1478–83. 10.1161/ATVBAHA.113.300168. 23766387

[29] Mantovani A , Sica A , Sozzani S , Allavena P , Vecchi A , Locati M . The chemokine system in diverse forms of macrophage activation and polarization. Trends Immunol. 2004; 25:677–86. 10.1016/j.it.2004.09.015. 15530839

[30] Lahmar Q , Keirsse J , Laoui D , Movahedi K , Van Overmeire E , Van Ginderachter JA . Tissue-resident versus monocyte-derived macrophages in the tumor microenvironment. Biochim Biophys Acta. 2016; 1865:23–34. 10.1016/j.bbcan.2015.06.009. 26145884

[31] Gomez Perdiguero E , Klapproth K , Schulz C , Busch K , Azzoni E , Crozet L , Garner H , Trouillet C , de Bruijn MF , Geissmann F , Rodewald HR . Tissue-resident macrophages originate from yolk-sac-derived erythro-myeloid progenitors. Nature. 2015; 518:547–51. 10.1038/nature13989. 25470051 PMC5997177

[32] Guilliams M , Scott CL . Does niche competition determine the origin of tissue-resident macrophages? Nat Rev Immunol. 2017; 17:451–60. 10.1038/nri.2017.42. 28461703

[33] Beattie L , Sawtell A , Mann J , Frame TC , Teal B , de Labastida Rivera F , Brown N , Walwyn-Brown K , Moore JW , MacDonald S , Lim EK , Dalton JE , Engwerda CR , et al. Bone marrow-derived and resident liver macrophages display unique transcriptomic signatures but similar biological functions. J Hepatol. 2016; 65:758–68. 10.1016/j.jhep.2016.05.037. 27262757 PMC5028381

[34] Reid DT , Reyes JL , McDonald BA , Vo T , Reimer RA , Eksteen B . Kupffer Cells Undergo Fundamental Changes during the Development of Experimental NASH and Are Critical in Initiating Liver Damage and Inflammation. PLoS One. 2016; 11:e0159524. 10.1371/journal.pone.0159524. 27454866 PMC4959686

[35] Krenkel O , Puengel T , Govaere O , Abdallah AT , Mossanen JC , Kohlhepp M , Liepelt A , Lefebvre E , Luedde T , Hellerbrand C , Weiskirchen R , Longerich T , Costa IG , et al. Therapeutic inhibition of inflammatory monocyte recruitment reduces steatohepatitis and liver fibrosis. Hepatology. 2018; 67:1270–83. 10.1002/hep.29544. 28940700

[36] Devisscher L , Verhelst X , Colle I , Van Vlierberghe H , Geerts A . The role of macrophages in obesity-driven chronic liver disease. J Leukoc Biol. 2016; 99:693–8. 10.1189/jlb.5RU0116-016R. 26936934

[37] Bain CC , Hawley CA , Garner H , Scott CL , Schridde A , Steers NJ , Mack M , Joshi A , Guilliams M , Mowat AM , Geissmann F , Jenkins SJ . Long-lived self-renewing bone marrow-derived macrophages displace embryo-derived cells to inhabit adult serous cavities. Nat Commun. 2016; 7:ncomms11852. 10.1038/ncomms11852. 27292029 PMC4910019

[38] Scott CL , Zheng F , De Baetselier P , Martens L , Saeys Y , De Prijck S , Lippens S , Abels C , Schoonooghe S , Raes G , Devoogdt N , Lambrecht BN , Beschin A , Guilliams M . Bone marrow-derived monocytes give rise to self-renewing and fully differentiated Kupffer cells. Nat Commun. 2016; 7:10321. 10.1038/ncomms10321. 26813785 PMC4737801

[39] Ju C , Tacke F . Hepatic macrophages in homeostasis and liver diseases: from pathogenesis to novel therapeutic strategies. Cell Mol Immunol. 2016; 13:316–27. 10.1038/cmi.2015.104. 26908374 PMC4856798

[40] Krenkel O , Tacke F . Liver macrophages in tissue homeostasis and disease. Nat Rev Immunol. 2017; 17:306–21. 10.1038/nri.2017.11. 28317925

[41] Lefere S , Degroote H , Van Vlierberghe H , Devisscher L . Unveiling the depletion of Kupffer cells in experimental hepatocarcinogenesis through liver macrophage subtype-specific markers. J Hepatol. 2019; 71:631–3. 10.1016/j.jhep.2019.03.016. 31213365

[42] Tacke F . Targeting hepatic macrophages to treat liver diseases. J Hepatol. 2017; 66:1300–12. 10.1016/j.jhep.2017.02.026. 28267621

[43] Zhang CH , Xu GL , Jia WD , Ge YS , Wang W . Can hepatocellular carcinoma be treated by Yondelis through targeting both tumor cells and tumor-associated macrophages? Hepatogastroenterology. 2010; 57:114–6. 20422884

[44] Deng YR , Liu WB , Lian ZX , Li X , Hou X . Sorafenib inhibits macrophage-mediated epithelial-mesenchymal transition in hepatocellular carcinoma. Oncotarget. 2016; 7:38292–305. 10.18632/oncotarget.9438. 27203677 PMC5122390

[45] Tang X , Mo C , Wang Y , Wei D , Xiao H . Anti-tumour strategies aiming to target tumour-associated macrophages. Immunology. 2013; 138:93–104. 10.1111/imm.12023. . 23113570 PMC3575762

[46] Roderburg C , Wree A , Demir M , Schmelzle M , Tacke F . The role of the innate immune system in the development and treatment of hepatocellular carcinoma. Hepat Oncol. 2020; 7:HEP17. 10.2217/hep-2019-0007. 32273975 PMC7137177

[47] Heindryckx F , Mertens K , Charette N , Vandeghinste B , Casteleyn C , Van Steenkiste C , Slaets D , Libbrecht L , Staelens S , Starkel P , Geerts A , Colle I , Van Vlierberghe H . Kinetics of angiogenic changes in a new mouse model for hepatocellular carcinoma. Mol Cancer. 2010; 9:219. 10.1186/1476-4598-9-219. 20727157 PMC2936339

[48] Fujii M , Shibazaki Y , Wakamatsu K , Honda Y , Kawauchi Y , Suzuki K , Arumugam S , Watanabe K , Ichida T , Asakura H , Yoneyama H . A murine model for non-alcoholic steatohepatitis showing evidence of association between diabetes and hepatocellular carcinoma. Med Mol Morphol. 2013; 46:141–52. 10.1007/s00795-013-0016-1. 23430399

[49] Body-Malapel M , Dharancy S , Berrebi D , Louvet A , Hugot JP , Philpott DJ , Giovannini M , Chareyre F , Pages G , Gantier E , Girardin SE , Garcia I , Hudault S , et al. NOD2: a potential target for regulating liver injury. Lab Invest. 2008; 88:318–27. 10.1038/labinvest.3700716. 18227809

[50] Arias-Loste MT , Iruzubieta P , Puente A , Ramos D , Santa Cruz C , Estebanez A , Llerena S , Alonso-Martin C , San Segundo D , Alvarez L , Lopez Useros A , Fabrega E , Lopez-Hoyos M , Crespo J . Increased Expression Profile and Functionality of TLR6 in Peripheral Blood Mononuclear Cells and Hepatocytes of Morbidly Obese Patients with Non-Alcoholic Fatty Liver Disease. Int J Mol Sci. 2016; 17:E1878. 10.3390/ijms17111878. 27834919 PMC5133878

[51] Laoui D , Van Overmeire E , Movahedi K , Van den Bossche J , Schouppe E , Mommer C , Nikolaou A , Morias Y , De Baetselier P , Van Ginderachter JA . Mononuclear phagocyte heterogeneity in cancer: different subsets and activation states reaching out at the tumor site. Immunobiology. 2011; 216:1192–202. 10.1016/j.imbio.2011.06.007. 21803441

[52] Elliott LA , Doherty GA , Sheahan K , Ryan EJ . Human Tumor-Infiltrating Myeloid Cells: Phenotypic and Functional Diversity. Front Immunol. 2017; 8:86. 10.3389/fimmu.2017.00086. 28220123 PMC5292650

[53] Lefere S , Puengel T , Hundertmark J , Penners C , Frank AK , Guillot A , de Muynck K , Heymann F , Adarbes V , Defrene E , Estivalet C , Geerts A , Devisscher L , et al. Differential effects of selective- and pan-PPAR agonists on experimental steatohepatitis and hepatic macrophages. J Hepatol. 2020; 73:757–70. 10.1016/j.jhep.2020.04.025. 32360434

[54] Ning G , Huang YL , Zhen LM , Xu WX , Li XJ , Wu LN , Liu Y , Xie C , Peng L . Prognostic Value of Complement Component 2 and Its Correlation with Immune Infiltrates in Hepatocellular Carcinoma. Biomed Res Int. 2020; 2020:3765937. 10.1155/2020/3765937. 32626741 PMC7312969

[55] Krenkel O , Hundertmark J , Abdallah AT , Kohlhepp M , Puengel T , Roth T , Branco DPP , Mossanen JC , Luedde T , Trautwein C , Costa IG , Tacke F . Myeloid cells in liver and bone marrow acquire a functionally distinct inflammatory phenotype during obesity-related steatohepatitis. Gut. 2020; 69:551–63. 10.1136/gutjnl-2019-318382. 31076404

[56] Patel P , Schutzer SE , Pyrsopoulos N . Immunobiology of hepatocarcinogenesis: Ways to go or almost there? World J Gastrointest Pathophysiol. 2016; 7:242–55. 10.4291/wjgp.v7.i3.242. 27574562 PMC4981764

[57] Prieto J , Melero I , Sangro B . Immunological landscape and immunotherapy of hepatocellular carcinoma. Nat Rev Gastroenterol Hepatol. 2015; 12:681–700. 10.1038/nrgastro.2015.173. 26484443

[58] Naugler WE , Sakurai T , Kim S , Maeda S , Kim K , Elsharkawy AM , Karin M . Gender disparity in liver cancer due to sex differences in MyD88-dependent IL-6 production. Science. 2007; 317:121–4. 10.1126/science.1140485. 17615358

[59] Wan S , Kuo N , Kryczek I , Zou W , Welling TH . Myeloid cells in hepatocellular carcinoma. Hepatology. 2015; 62:1304–12. 10.1002/hep.27867. 25914264 PMC4589430

[60] Wu J , Li J , Salcedo R , Mivechi NF , Trinchieri G , Horuzsko A . The proinflammatory myeloid cell receptor TREM-1 controls Kupffer cell activation and development of hepatocellular carcinoma. Cancer Res. 2012; 72:3977–86. 10.1158/0008-5472.CAN-12-0938. 22719066 PMC3694446

[61] Lurier EB , Dalton D , Dampier W , Raman P , Nassiri S , Ferraro NM , Rajagopalan R , Sarmady M , Spiller KL . Transcriptome analysis of IL-10-stimulated (M2c) macrophages by next-generation sequencing. Immunobiology. 2017; 222:847–56. 10.1016/j.imbio.2017.02.006. 28318799 PMC5719494

[62] Bleriot C , Dupuis T , Jouvion G , Eberl G , Disson O , Lecuit M . Liver-resident macrophage necroptosis orchestrates type 1 microbicidal inflammation and type-2-mediated tissue repair during bacterial infection. Immunity. 2015; 42:145–58. 10.1016/j.immuni.2014.12.020. 25577440

[63] Zigmond E , Samia-Grinberg S , Pasmanik-Chor M , Brazowski E , Shibolet O , Halpern Z , Varol C . Infiltrating monocyte-derived macrophages and resident kupffer cells display different ontogeny and functions in acute liver injury. J Immunol. 2014; 193:344–53. 10.4049/jimmunol.1400574. 24890723

[64] Devisscher L , Scott CL , Lefere S , Raevens S , Bogaerts E , Paridaens A , Verhelst X , Geerts A , Guilliams M , Van Vlierberghe H . Non-alcoholic steatohepatitis induces transient changes within the liver macrophage pool. Cell Immunol. 2017; 322:74–83. 10.1016/j.cellimm.2017.10.006. 29111158

[65] Morias Y , Abels C , Laoui D , Van Overmeire E , Guilliams M , Schouppe E , Tacke F , deVries CJ , De Baetselier P , Beschin A . Ly6C- Monocytes Regulate Parasite-Induced Liver Inflammation by Inducing the Differentiation of Pathogenic Ly6C+ Monocytes into Macrophages. PLoS Pathog. 2015; 11:e1004873. 10.1371/journal.ppat.1004873. 26020782 PMC4447383

[66] Dal-Secco D , Wang J , Zeng Z , Kolaczkowska E , Wong CH , Petri B , Ransohoff RM , Charo IF , Jenne CN , Kubes P . A dynamic spectrum of monocytes arising from the *in situ* reprogramming of CCR2+ monocytes at a site of sterile injury. J Exp Med. 2015; 212:447–56. 10.1084/jem.20141539. 25800956 PMC4387291

[67] David BA , Rezende RM , Antunes MM , Santos MM , Freitas Lopes MA , Diniz AB , Sousa Pereira RV , Marchesi SC , Alvarenga DM , Nakagaki BN , Araujo AM , Dos Reis DS , Rocha RM , et al. Combination of Mass Cytometry and Imaging Analysis Reveals Origin, Location, and Functional Repopulation of Liver Myeloid Cells in Mice. Gastroenterology. 2016; 151:1176–91. 10.1053/j.gastro.2016.08.024. 27569723

[68] Borst K , Frenz T , Spanier J , Tegtmeyer PK , Chhatbar C , Skerra J , Ghita L , Namineni S , Lienenklaus S , Koster M , Heikenwaelder M , Sutter G , Kalinke U . Type I interferon receptor signaling delays Kupffer cell replenishment during acute fulminant viral hepatitis. J Hepatol. 2018; 68:682–90. 10.1016/j.jhep.2017.11.029. 29274730

[69] Bonnardel J , T’Jonck W , Gaublomme D , Browaeys R , Scott CL , Martens L , Vanneste B , De Prijck S , Nedospasov SA , Kremer A , Van Hamme E , Borghgraef P , Toussaint W , et al. Stellate Cells, Hepatocytes, and Endothelial Cells Imprint the Kupffer Cell Identity on Monocytes Colonizing the Liver Macrophage Niche. Immunity. 2019; 51:638–54.e9. 10.1016/j.immuni.2019.08.017. 31561945 PMC6876284

[70] Novikova MV , Khromova NV , Kopnin PB . Components of the Hepatocellular Carcinoma Microenvironment and Their Role in Tumor Progression. Biochemistry (Mosc). 2017; 82:861–73. 10.1134/S0006297917080016. 28941454

[71] Van Campenhout S , Tilleman L , Lefere S , Vandierendonck A , Raevens S , Verhelst X , Geerts A , Van Nieuwerburgh F , Van Vlierberghe H , Devisscher L . Myeloid-specific IRE1alpha deletion reduces tumour development in a diabetic, non-alcoholic steatohepatitis-induced hepatocellular carcinoma mouse model. Metabolism. 2020; 107:154220. 10.1016/j.metabol.2020.154220. 32243868

[72] Ostuni R , Kratochvill F , Murray PJ , Natoli G . Macrophages and cancer: from mechanisms to therapeutic implications. Trends Immunol. 2015; 36:229–39. 10.1016/j.it.2015.02.004. 25770924

[73] Yang JD , Nakamura I , Roberts LR . The tumor microenvironment in hepatocellular carcinoma: current status and therapeutic targets. Semin Cancer Biol. 2011; 21:35–43. 10.1016/j.semcancer.2010.10.007. 20946957 PMC3050428

[74] Aravalli RN . Role of innate immunity in the development of hepatocellular carcinoma. World J Gastroenterol. 2013; 19:7500–14. 10.3748/wjg.v19.i43.7500. 24282342 PMC3837249

[75] Lefere S , Tacke F . Macrophages in obesity and non-alcoholic fatty liver disease: Crosstalk with metabolism. JHEP Rep. 2019; 1:30–43. 10.1016/j.jhepr.2019.02.004. 32149275 PMC7052781

[76] Guillot A , Tacke F . Liver Macrophages: Old Dogmas and New Insights. Hepatol Commun. 2019; 3:730–43. 10.1002/hep4.1356. 31168508 PMC6545867

[77] Ingersoll MA , Spanbroek R , Lottaz C , Gautier EL , Frankenberger M , Hoffmann R , Lang R , Haniffa M , Collin M , Tacke F , Habenicht AJ , Ziegler-Heitbrock L , Randolph GJ . Comparison of gene expression profiles between human and mouse monocyte subsets. Blood. 2010; 115:e10–9. 10.1182/blood-2009-07-235028. 19965649 PMC2810986

[78] Kleiner DE , Makhlouf HR . Histology of Nonalcoholic Fatty Liver Disease and Nonalcoholic Steatohepatitis in Adults and Children. Clin Liver Dis. 2016; 20:293–312. 10.1016/j.cld.2015.10.011. 27063270 PMC4829204

