# Supplementary material for: Characterization of the inflammatory microenvironment and hepatic macrophage subsets in experimental hepatocellular carcinoma models
Source: Oncotarget. 2021 Mar 16;12(6):562–77. doi: 10.18632/oncotarget.27906 (PMC7984829; doi:10.18632/oncotarget.27906)
Supplement: Supplementary file 1 [file oncotarget-12-562-s001.pdf]

Characterization of the inflammatory microenvironment and hepatic macrophage subsets in experimental hepatocellular carcinoma models

SUPPLEMENTARY MATERIALS

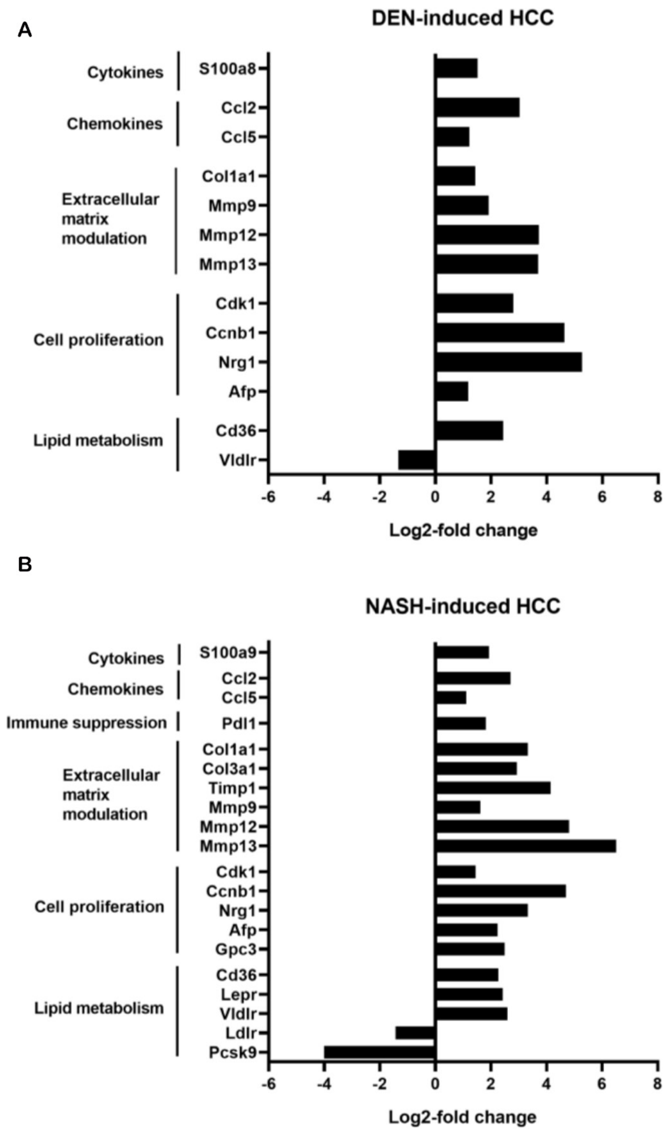

Supplementary Figure 1: Differential gene expression in full liver tissue in (**A**) DEN-induced HCC and (**B**) NASH-induced HCC, comparing control (n = 4) and intermediate stage (n = 4) HCC development.

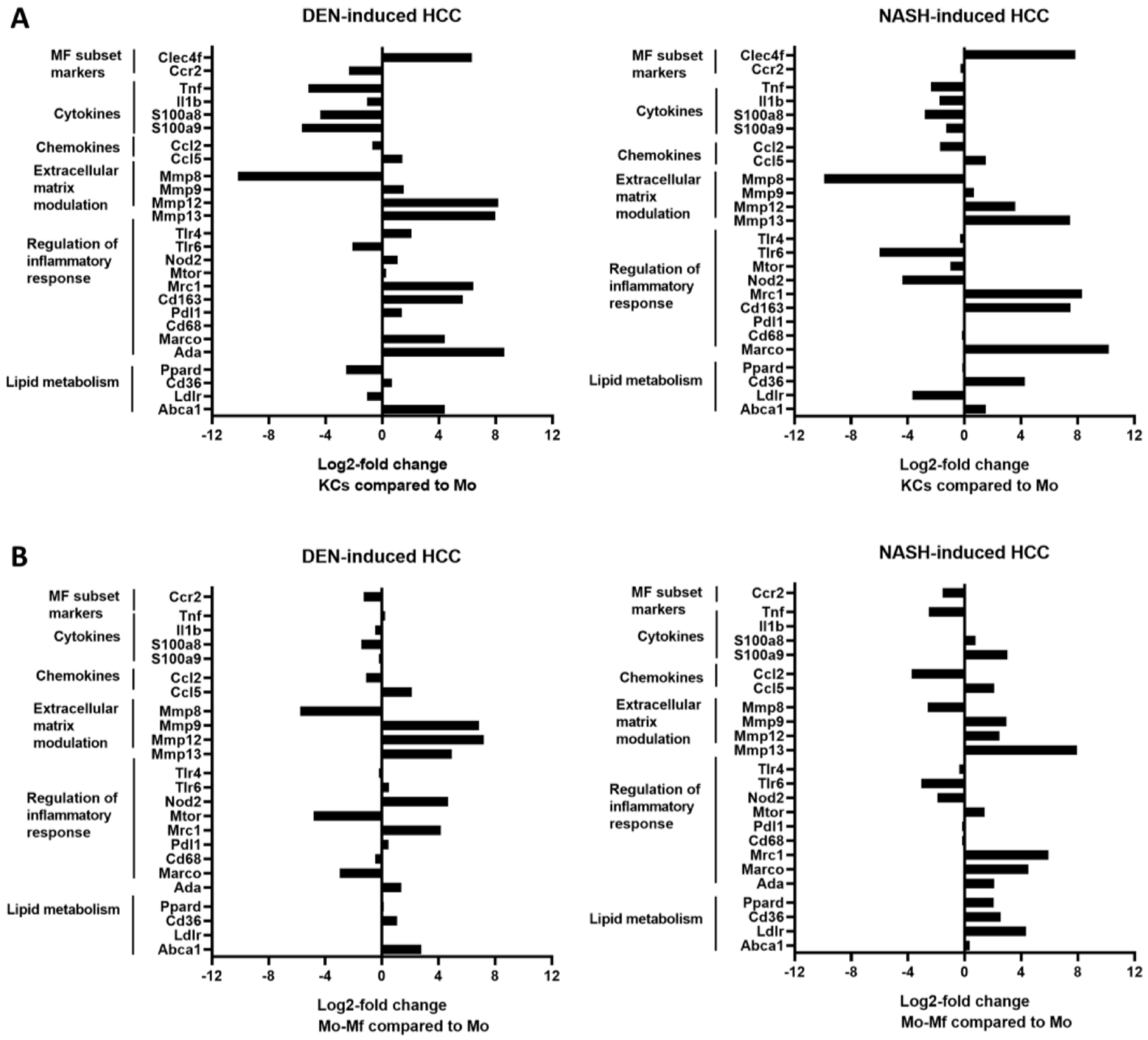

Supplementary Figure 2: Differential gene expression in (**A**) KCs compared to Mo and (**B**) Mo-Mf compared to Mo at end-stage HCC development in DEN-induced HCC (*n* = 4) and NASH-induced HCC model (*n* = 4).
